# Polymorphism in the Promoter Region of NFE2L2 Gene Is a Genetic Marker of Susceptibility to Cirrhosis Associated with Alcohol Abuse

**DOI:** 10.3390/ijms20143589

**Published:** 2019-07-23

**Authors:** Kemper Nunes dos Santos, Rodrigo M. Florentino, Andressa França, Antônio Carlos Melo Lima Filho, Marcone Loiola dos Santos, Dabny Missiaggia, Matheus de Castro Fonseca, Igor Brasil Costa, Paula Vieira Teixeira Vidigal, Michael H. Nathanson, Fernanda de Oliveira Lemos, M. Fatima Leite

**Affiliations:** 1 Universidade Federal de Minas Gerais, Belo Horizonte, MG 31270-901, Brazil; 2 Laboratório Nacional de Biociências (LNBio), Centro de Pesquisa em Energia e Materiais (CNPEM), Campinas, SP 13083-970, Brazil; 3 Instituto de Pesquisas Evandro Chagas – IEC, Ananindeua, PA 67030-000, Brazil; 4 Section of Digestive Diseases, Yale University School of Medicine, New Haven, CT 06510, USA

**Keywords:** Alcoholic liver disease, Nrf2, Polymorphism, NFE2L2 gene

## Abstract

Alcoholic liver disease (ALD) is a highly prevalent spectrum of pathologies caused by alcohol overconsumption. Morbidity and mortality related to ALD are increasing worldwide, thereby demanding strategies for early diagnosis and detection of ALD predisposition. A potential candidate as a marker for ALD susceptibility is the transcription factor nuclear factor erythroid-related factor 2 (Nrf2), codified by the nuclear factor erythroid 2-related factor 2 gene (NFE2L2). Nrf2 regulates expression of proteins that protect against oxidative stress and inflammation caused by alcohol overconsumption. Here, we assessed genetic variants of NFE2L2 for association with ALD. Specimens from patients diagnosed with cirrhosis caused by ALD were genotyped for three NFE2L2 single nucleotide polymorphisms (SNP) (SNPs: rs35652124, rs4893819, and rs6721961). Hematoxylin & eosin and immunohistochemistry were performed to determine the inflammatory score and Nrf2 expression, respectively. SNPs rs4893819 and rs6721961 were not specifically associated with ALD, but analysis of SNP rs35652124 suggested that this polymorphism predisposes to ALD. Furthermore, SNP rs35652124 was associated with a lower level of Nrf2 expression. Moreover, liver samples from ALD patients with this polymorphism displayed more severe inflammatory activity. Together, these findings provide evidence that the SNP rs35652124 variation in the Nrf2-encoding gene NFE2L2 is a potential genetic marker for susceptibility to ALD.

## 1. Introduction

Alcoholic liver disease (ALD), a spectrum of pathologies that include fatty liver disease, alcoholic hepatitis, and cirrhosis caused by alcohol overconsumption [1,2], is a global public health issue. Approximately 2.4 billion people consume alcohol worldwide, and 75 million people are at risk of alcohol-associated liver disease [3]. It is estimated that 2 million people die of liver disease each year, and up to 50% of these cases are due in part to alcoholic cirrhosis [1]. ALD, as with other chronic liver diseases, may not be clinically apparent early in the disease process. This highlights the importance of early diagnosis for management and treatment to prevent cirrhosis-related morbidity and mortality, as well as to detect ALD susceptibility.

Ethanol metabolism generates reactive oxygen species (ROS), causing oxidative stress and inflammation, the main pathogenic events involved in ALD [4,5]. Therefore, preventing the increase in ROS is a potential mechanism to protect the liver tissue from the damage caused by alcohol consumption. An endogenous mechanism of hepatoprotection is mediated by the nuclear erythroid-related factor 2 (Nrf2). Nrf2 is a basic leucine zipper transcription factor that controls the expression of the antioxidant response element (ARE)—dependent genes, leading to the expression of cytoprotective and anti-inflammatory proteins [6,7]. Dysregulation of Nrf2 activity correlates with the development of several chronic inflammatory diseases [7,8,9,10]. For instance, knock-out (KO) mice for Nrf2 displayed aggravated liver injury after alcohol ingestion [11,12].

Progression and prognosis of alcohol-related liver disease is influenced by multiple factors, including genetics [2]. The most common type of genetic variation is the single nucleotide polymorphism (SNP), which represents a nucleotide substitution in the genome that is associated with altered vulnerability to disease [13,14]. SNPs in the promoter region of the Nuclear factor erythroid 2-related factor 2 gene (NFE2L2) have been associated with disease susceptibility and other inherited phenotypes, especially conditions characterized by increased levels of oxidative stress [15]. However, SNPs in NFE2L2 have not yet been related to ALD. Here we investigated whether polymorphisms in the promoter region of the NFE2L2 gene are associated with worse prognosis in a cohort of ALD patients. For comparison, we examined the specificity of NFE2L2 SNPs for ALD by comparing the occurrence of these SNPs in patients with chronic hepatitis C virus (HCV) infection. Our findings suggest SNP rs35652124 is a specific genetic marker for susceptibility to the development of ALD. 

## 2. Results

### 2.1. Single Nucleotide Polymorphism at -274 of the NFE2L2 Promoter Region Is a Genetic Marker for ALD Susceptibility

**Figure 1:**
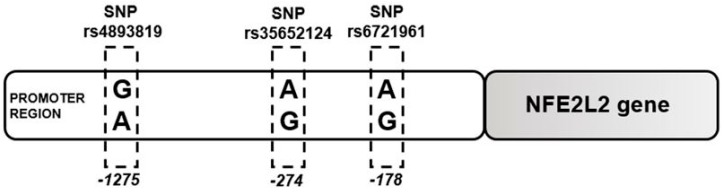
Schematic position of the selected polymorphisms in nuclear factor erythroid 2-related factor 2 gene (NFE2L2). The three polymorphisms studied in this work are localized in promoter region of NFE2L2 gene. Single Nucleotide Polymorphism (SNP) rs6721961 is located at position -178, where can be present an adenine (A) or guanine (G). SNP rs35652124 is in position -274 in the promoter region, and the variation is the same of the first. Lastly, SNP rs4893819 is localized in position -1275 in the promoter region of NFE2L2 gene. In this position, the variation occurs between G and A. All these positions are considering the position in relation 3’ → 5’.

Two of the three analyzed polymorphisms in the promoter region of NFE2L2, the gene that encodes Nrf2 (Figure 1), showed clinical significance in ALD samples. 

The SNP rs35652124 analysis showed the polymorphism -274 adenine (A) in the promoter region of NFE2L2 gene in most ALD patients (n = 55 patients (59.8%)), while the variant -274 guanine (G) was also present but less frequently in the same group (n = 37 patients (40.2%)). Conversely, control group showed the variant -274G in most of the samples (n = 25 patients (59.5%)), whereas the polymorphism -274A was less frequent in normal tissue (n = 17 patients (40.5%)) (Table 1). We also performed the SNP rs35652124 analysis in patients with cirrhosis caused by HCV to evaluate the specificity of this polymorphism for ALD. In contrast to what was observed in ALD, most patients with cirrhosis caused by HCV had the variant -274G (n = 19 patients - 67.9%) instead of -274A (n = 9 patients—32.1%) (Table 2). The comparison between HCV and control liver samples did not show any significant difference regarding the frequency of the variant -274A or -274G (Table 3). These observations are consistent with the idea that the presence of an A in SNP rs35652124 is associated with ALD development, while the presence of a G may be a protective factor.

The second polymorphism with clinical importance was the SNP rs6721961, located at position -178 in the promoter region of the NFE2L2 gene. The frequency of -178A and -178G in the samples from ALD patients was not different when we compared them to HCV samples (HCV—A: n = 22 patients—61.1%; and G: n = 14 patients—38.9%/ ALD A: n = 38 patients—41.3%; and G: n = 54 patients—58.7%) (Table 4). However, the frequency of the alleles A/A and A/G was lower in the ALD group (allele A/A: n = 27 patients—58.7%; and allele G/G: n = 19 patients—41.3%) than in the HCV group (alleles A/A and A/G: n = 16 patients—88.9%; allele G/G: n = 2 patients—11.1%) (Table 4). Together, these results suggest that the presence of the nucleotide A in the position -178 of the promoter region of NFE2L2 is associated with cirrhotic liver disease, since both ALD and HCV samples preferentially showed this polymorphism.

The third polymorphism analyzed was the SNP rs4893819, in the position -1275 of the promoter region of NFE2L2 gene. The comparison between ALD and HCV liver samples did not show any significant difference regarding the frequency of the variant -1275A or -1275G (Table 5). 

Together, these results are consistent with the idea that the polymorphism -274A in the promoter region of NFE2L2 predisposes to ALD while the polymorphism at -178A might be associated with susceptibility to develop cirrhosis from ALD plus other causes as well.

### 2.2. Single Nucleotide Polymorphism Rs35652124 in the NFE2L2 Gene Correlates With Inflammatory Score in ALD

**Figure 2:**
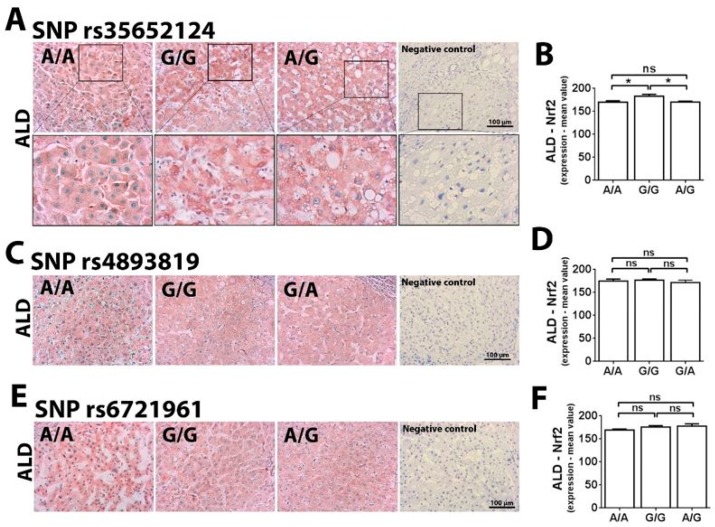
Nrf2 expression in the different polymorphisms of NFE2L2 gene in patients with ALD-associated cirrhosis. (**A**) Immunohistochemistry for Nrf2 in in the SNP rs35652124 genotypes. (**B**) Quantification of Nrf2 expression (mean value) in the different genotypes of SNP rs35652124. (**C**) Immunohistochemistry for Nrf2 in the SNP rs4893819 genotypes. (**D**) Quantification of Nrf2 expression (mean value) in the different genotypes of SNP rs4893819. (**E**) Immunohistochemistry for Nrf2 in the SNP rs6721961 genotypes. (**F**) Quantification of Nrf2 expression (mean value) in different genotypes of SNP rs6721961 (n = 15 patients with ALD-associated cirrhosis; and n = 5 patients in each genotype. * *p* < 0.05. Values are expressed as mean ± SEM).

In light of the protective effect of Nrf2 against oxidative stress caused by alcohol consumption, we aimed to assess whether polymorphisms in the promoter region of NFE2L2 directly correlate with expression of Nrf2 in the liver. To address this question, immunohistochemistry was performed in the ALD and HCV samples after Nrf2 antibody optimization, using liver specimens from healthy patients (negative control—nonspecific binding), and specimens from patients with extrahepatic biliary atresia (positive control for Nrf2 nuclear translocation) [16] (Figure S1). Although Nrf2 staining was not nuclear in most ALD specimens analyzed, Nrf2 was localized throughout the cytoplasm in the ALD samples, and preferentially in the perinuclear region (Figure 2). This altered distribution could be due to the fact that ALD specimens used here were from patients with at least six months of alcohol abstinence before liver transplantation. Therefore, the absence of the acute alcohol stimuli could contribute to the lower nuclear translocation of Nrf2 observed in these ALD samples. In addition to the Nrf2 nuclear translocation, the de novo synthesis of this transcription factor is also involved in the mechanism of cell protection after exposure to reactive oxygen species [17,18]. This is in agreement with our findings that in SNP rs35652124 polymorphisms, Nrf2 expression was higher in samples exhibiting the -274G/G allele than in samples with the -274A/A or -274A/G alleles (G/G = 183.1 ± 3.9; A/A = 170.1 ± 2.9; A/G = 70.3 ± 1.9), (Figure 2A,B). The expression of superoxide dismutase 1 (SOD1), a gene which transcription is targeted for a variety of transcription factors, including Nrf2 [19], was also observed in most ALD samples (Figure S2), suggesting some level of Nrf2 activation. These results support the observation that the presence of -274A in SNP rs35652124, both in homozygosity (A/A) or heterozygosity (A/G), may be a risk factor for ALD, while the -274G/G allele is a protective factor.

When it comes to the SNP rs4893819 (Figure 2C,D) and SNP rs6721961 (Figure 2E,F) polymorphisms, no correlation was observed regarding Nrf2 expression and the alleles A/A, A/G or G/G. Moreover, Nrf2 expression was not associated with the presence of any specific allele in SNP rs35652124, SNP rs4893819 or SNP rs6721961 in HCV samples (Figure S3).

There was no correlation between the polymorphism in the promoter region of NFE2L2 and the biochemical data of patients with either ALD (Table 6) or HCV (Tables S2–S4). These results might reflect the end-stage nature of the ALD and HCV cases, because all these patients had cirrhosis and required liver transplantation. 

**Figure 3:**
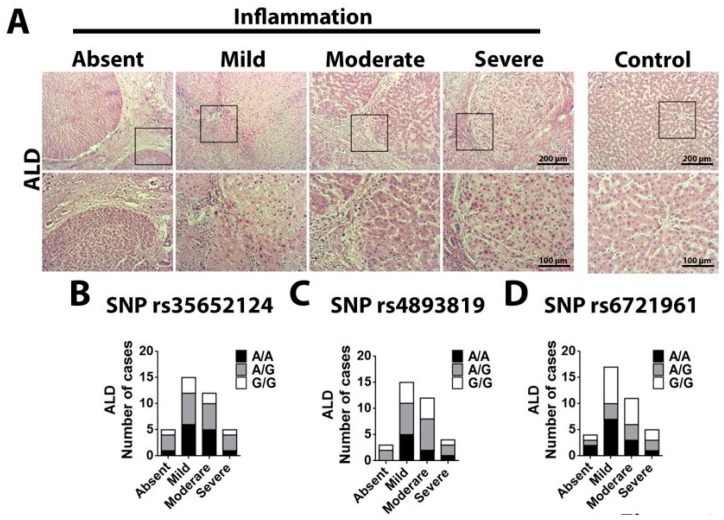
Inflammation score in the different polymorphism of NFE2L2 gene in patients with cirrhosis from ALD. (**A**) HE from ALD patients showing the score of inflammation. The METAVIR parameters are considered: A0 mean no inflammation activity; A1 means mild inflammatory activity; A2 means moderate inflammatory activity and A3 means intense inflammatory activity. (**B**) Number of cases in each inflammatory score considering the polymorphism SNP rs35652124. (**C**) Number of cases in each inflammatory score considering the polymorphism SNP rs4893819. (**D**) Number of cases in each inflammatory score considering the polymorphism SNP rs6721961 (n = 37 patients for SNP rs35652124; n = 37 patients for SNP rs6721961; and n = 34 patients for SNP rs4893819).

Finally, the association between histological findings and polymorphisms in the promoter region of NFE2L2 was evaluated after histological analysis of the hematoxylin and eosin (H&E)-stained ALD samples. Chronic liver disease can be divided in different stages of fibrosis levels and inflammatory activity [20]. All the ALD and HCV liver samples showed higher level of fibrosis, in such a way that just the inflammatory activity was scored (Figure 3A). For the SNP rs35652124 in the ALD samples (Figure 3B), we observed higher inflammatory activity for the alleles -274A/A and -274A/G (A/A: absent = 1 case; mild = 6 cases; moderate = 5 cases; severe = 1 case. A/G: absent = 3 cases; mild = 6 cases; moderate = 5 cases; severe = 3 cases). On the other hand, most of the samples with -274G/G allele showed attenuated inflammatory activity (G/G: absent = 1 case; mild = 3 cases; moderate = 2 cases; severe = 1 case). For the SNP rs6721961 -178A allele as a risk factor for liver damage, no difference was observed in the inflammatory activity (A/A: absent = 1 case; mild = 3 cases; moderate = 3 cases; severe = 2 case. A/G: absent = 2 cases; mild = 7 cases; moderate = 3 cases; severe = 1 cases. G/G: absent = 1 case; mild = 7 cases; moderate = 5 cases; severe = 2 case) (Figure 3C). The absence of correlation between the polymorphism and inflammatory activity was also observed for the SNP rs4893819 (A/A: absent = 0 case; mild = 5 cases; moderate = 2 cases; severe = 1 case. G/A: absent = 1 cases; mild = 4 cases; moderate = 4 cases; severe = 1 cases. G/G: absent = 2 case; mild = 6 cases; moderate = 6 cases; severe = 2 cases) (Figure 3D).

Together, these results suggest that polymorphism -274A in the promoter region of NEF2L2 gene is associated with worse inflammatory activity in ALD samples.

## 3. Discussion

Single nucleotide polymorphisms are the most common type of polymorphisms and occur at a frequency of approximately 1 in 1000 base pairs [21] throughout the genome (coding sequences, intronic sequences, and promoter region). 

In this study, we established, for the first time, a specific and functional association between a SNP in the promoter region of the NFE2L2 gene and susceptibility to ALD. ALD can lead to cirrhosis, liver cancer, and liver failure, and is the most frequent indication for orthotopic liver transplantation in some populations [22]. Although the pathogenesis of ALD has not yet been fully elucidated, an interaction among behavioral, environmental and genetic factors is appreciated [22,23]. Most studies of the genetic predisposition to ALD have focused on genes related to cell metabolism and detoxifying enzymes [24,25,26]. Recently, genes for patatin-like phospholipase domain-containing protein 3 (PNPLA3), transmembrane 6 superfamily member 2 (TM6SF2) and membrane-bound O-acyltransferase domain-containing 7 (MBOAT7) have emerged as potential markers of susceptibility to develop alcohol-related liver injury [27]. These three proteins are involved in lipid metabolism, and therefore may also participate in the pathogenesis of ALD, because steatosis is the initial histological finding from alcohol overconsumption [27]. For example, PNPLA3 is expressed in hepatocytes, and functions as a lipase, catalyzing the hydrolysis of triglycerides [28]. SNP rs738409 in the PNPLA3 gene is strongly associated with the alcoholic hepatitis [29,30], alcoholic cirrhosis [31,32], hepatocellular carcinoma (HCC) and reduced transplantation-free survival [32,33,34], as well as non-alcoholic fatty liver disease (NAFLD) [27,35]. TM6SF2 is involved in very low-density lipoprotein (VLDL) secretion, and the rs58542926 variant has been associated with ALD cirrhosis [31], HCC [34], and NAFLD [36,37]. SNP rs10401969 of the TM6SF2 gene also correlates with ALD cirrhosis [31]. MBOAT7 catalyzes the transfer of fatty acids between phospholipids and lysophospholipids, and the SNP rs641738 is associated with higher risk of NAFLD [38] and inflammation and fibrosis in chronic hepatitis B [39], while rs626283 is correlated with ALD cirrhosis [31]. 

Generation of ROS is a common consequence of alcohol metabolism [23,40], which is counteracted by the Nrf2/ARE axis [41]. Nrf2 is a switch for the endogenous antioxidant response by activating its downstream target genes responsible for regulating oxidative stress and inactivating toxic chemicals and proteins [9,42,43,44]. Although a decline in Nrf2 expression has already been observed in older rodents [45] and in age-related neurodegenerative disorder in humans [46], which can contribute to the susceptibility to various diseases, the specimens selected for the current work were from patients with similar ages. Several endogenous mechanisms are responsible for reducing cellular oxidative stress through expression of antioxidant genes, such as glutathione-S-transferase (GST), coenzyme Q10 (Q10), NAD(P)Hiquinone oxidoreductase (QR), and superoxide dismutase 1 (SOD1) which have the common promoter element called the antioxidant response element [47]. Activation of Nrf2 as a response to cell injury has already been described in different liver cells and several liver diseases, such as cholestatic liver injury [16], viral hepatitis, drug-induced hepatitis, liver fibrosis, and cirrhosis, HCC, and alcoholic and non-alcoholic steatohepatitis [44]. For instance, impairment of acetaldehyde detoxification, aggravation of inflammatory response, and liver failure are key common events observed in ethanol-fed Nrf2 knock-out mice, confirming the importance of this transcriptional factor to prevent liver injury [12]. 

Several SNPs in the encoding region of the NFE2L2 gene, have already been associated with diseases characterized by oxidative stress and inflammation, including acute lung injury [48], chronic gastritis [49], gastric ulcer [50], ulcerative colitis [51], chronic obstructive pulmonary disease [52], and type 2 diabetes mellitus [15]. Our findings suggest that a variant of the NF2L2 gene specifically correlates with ALD cirrhosis. Our findings show that the variant -274A in SNP rs35652124 correlates with the occurrence of ALD cirrhosis but not with HCV-associated cirrhosis. Additionally, we observed that this variant associates with reduced expression of Nrf2, followed by a more severe inflammatory condition, which is in agreement with previous studies showing that Nrf2-deficient mice exhibit worst liver damage induced by alcohol intake [12]. This polymorphism of NFE2L2 has also been implicated in other diseases, such as Parkinson’s disease (PD) [53], hypertension, and cardiovascular disease [54]. In PD, for example, 10 SNPs within the NFE2L2 gene, including 3 exonic SNPs, 2 intronic SNPs, 3 promoter SNPs, and 2 SNPs in 3′ region of NFE2L2 gene were found [55].

In conclusion, our findings provide evidence that single nucleotide variations in the promoter region of NFE2L2, specifically the variant –274A of SNP rs35652124, might contribute to the pathogenesis of cirrhosis from ALD. In addition, the lower Nrf2 expression and severe inflammatory activity observed in patients carrying the -274A allele, suggest the potential relevance of this polymorphism in ALD susceptibility and in the progression of this disease

## 4. Materials and Methods 

### 4.1. Human Specimens

Human liver tissue specimens and clinical data from 49 patients with ALD in the cirrhotic phase were collected from patients, with alcohol abstinence for at least 6 months, who underwent liver transplantation at Hospital das Clínicas, Federal University of Minas Gerais (UFMG), from 1998 to 2015. Eighteen liver specimens from patients with HCV and 21 liver biopsies from liver organ donors, from 2012 to 2017, were used for comparison to the ALD group (Table S1). The average age of the patients used in this study were similar among the groups (control: 49.5, ALD: 53.6, and HCV: 57.9; p >0.05 one-way analysis of variance (ANOVA). The study was approved on 31st may 2017 by the Research Ethics Committee of UFMG, CAAE 67814117.6.0000.5149. Written informed consent was obtained from all subjects.

### 4.2. Genotyping

SNP analysis of the variants present in the promoter region of NFE2L2 gene was performed in the liver specimens of ALD, HCV, and healthy groups. SNPs rs35652124 (-214 A ã G), rs4893819 (-1275 G ã A) and rs6721961 (-178 A ã G) were analyzed in approximately 25 mg of paraffin-embedded hepatic tissues. Genomic DNA was extracted and purified with the DNeasy Blood & Tissue kit (Qiagen^®^, Hilden, North Rhine-Westphalia, Germany), in accordance with the manufacturer’s instruction. For genotyping, the genomic DNA was incubated with rhAmp™ Genotyping Master Mix and rhAmp™ Reporter Mix and the primers (rhAmp™ SNP Assay) for the SNPs rs35652124, rs4893819, and rs6721961, according to the guidelines (Integrated DNA Technologies—IDT^®^, Coralville, Iowa, USA). The polymerase chain reaction (PCR) amplification was performed in the 7500 real-time PCR system (Thermo Fisher Scientific, Foster City, California, USA) under the following conditions: 95 °C for 10 minutes (1 cycle), 40 cycles at 95 °C for 10 sec., 60 °C for 30 sec., and 68°C for 20 sec. The assay model was the rhAmp™ SNP Genotyping System, for qPCR-SNP (IDT^®^, Coralville, Iowa, USA).

### 4.3. Histopathological Analysis

Formalin-fixed, paraffin-embedded human liver specimens were sectioned at 4 µm thicknesses and stained with hematoxylin and eosin (H&E). The slides were evaluated under a light microscope (CX4; Olympus, Shinjuku, Tokyo, Japan) and the inflammatory cell infiltrate, polymorphonuclear leukocytes and mononuclear cells, in the epithelial and connective tissues was graded as absent—no inflammation activity; mild inflammatory activity; moderate inflammatory activity; and intense inflammatory activity, by an experienced pathologist, in a blinded fashion [20]. 

### 4.4. Immunohistochemistry

Immunohistochemistry was performed using formalin-fixed, paraffin-embedded human liver specimens with the Novolink Polymer Detection Kit (Leica Biosystems, Benton Lane, Newcastle, UK). Four µm-thick sections were dewaxed, and antigen retrieval was performed in citrate buffer 1 mM (pH 6.0). Following peroxidase and protein block, specimens were incubated with anti-Nfr2 (Abcam, Cambridge, MA, USA) and anti-SOD1 (Santa Cruz, Dallas, TX, USA) antibody overnight at room temperature. Reactions were revealed by applying 3,3’-diaminobenzidine (DAB). To compare Nfr2 expression in ALD, HCV, and healthy groups, images were converted to 8-bit. Regions of interest were selected and pixel intensity (0-255, grayscale) was measured in Image J software (Bethesda, Maryland, USA) [56].

### 4.5. Statistical Analysis

Results are presented as mean ± SEM. Data were analyzed using GraphPad Prism software (version 7; GraphPad Software, La Jolla, CA, USA). Differences among experimental groups were assessed for significance *p* < 0.05, using the Student’s t-test or one-way ANOVA followed by Bonferroni post-test.

## Figures and Tables

**Table 1:** Allele Frequencies Compared to Alcoholic liver disease (ALD)/Control Groups—SNP rs35652124 (-214 A > G).

Allele	ALD *n* (%)		Control *n* (%)		*p* Value
A/A	18	39.1	5	23.8	0.1457
G/G	9	19.6	9	42.9	
A/G	19	41.3	7	33.3	
**Total**	46	100.0	21	100	
A/A	18	39.1	5	23.8	0.2192
G/G_A/G	28	60.9	16	76.2	
**Total**	46	100.0	21	100	
A/A_A/G	37	80.4	12	57.1	**0.0460**
G/G	9	19.6	9	42.9	
**Total**	46	100.0	21	100	
A	55	59.8	17	40.5	**0.0387**
G	37	40.2	25	59.5	
**Total**	92	100.0	42	100	

**Table 2:** Allele Frequencies Compared to Alcoholic liver disease (ALD)/hepatitis C virus (HCV) Groups—SNP rs35652124 (-214 A > G).

Allele	ALD *n* (%)		HCV *n* (%)		*p* Value
A/A	18	39.1	2	14.3	0.0668
G/G	9	19.6	7	50.0	
A/G	19	41.3	5	35.7	
**Total**	46	100.0	14	100.0	
A/A	18	39.1	2	14.3	0.0746
G/G_A/G	28	60.9	12	85.7	
**Total**	46	100.0	14	100.0	
A/A_A/G	37	80.4	7	50.0	**0.0345**
G/G	9	19.6	7	50.0	
**Total**	46	100.0	14	100.0	
A	55	59.8	9	32.1	**0.0106**
G	37	40.2	19	67.9	
**Total**	92	100.0	28	100.0	

**Table 3:** Allele Frequencies Compared to hepatitis C virus (HCV)/Control Groups—SNP rs35652124 (-214 A > G).

Allele	HCV *n* (%)		Control *n* (%)		*p* Value
A/A	2	14.3	5	23.81	0.7898
G/G	7	50.0	9	42.86	
A/G	5	35.7	7	33.33	
**Total**	14	100.0	21	100	
A/A	2	14.3	5	23.81	0.4995
G/G_A/G	12	85.7	16	76.19	
**Total**	14	100.0	21	100	
A/A_A/G	7	50.0	12	57.14	0.6846
G/G	7	50.0	9	42.86	
**Total**	14	100.0	21	100	
A	9	32.1	17	40.48	0.4832
G	19	67.9	25	59.52	
**Total**	28	100.0	42	100	

**Table 4:** Allele Frequencies Compared to Alcoholic liver disease (ALD)/hepatitis C virus (HCV) Groups—SNP rs6721961 (-178 A > G).

Allele	ALD *n* (%)		HCV *n* (%)		*p* Value
A/G	16	34.8	10	55.6	0.0531
A/A	11	23.9	6	33.3	
G/G	19	41.3	2	11.1	
**Total**	46	100.0	18	100.0	
A/A	11	23.9	6	33.3	0.6510
A/G_G/G	35	76,1	12	66.7	
**Total**	46	100.0	18	100.0	
A/A_A/G	27	58.7	16	88.9	**0.0360**
G/G	19	41.3	2	11.1	
**Total**	46	100.0	18	100.0	
A	38	41.3	22	61.1	0.0685
G	54	58.7	14	38.9	
**Total**	92	100.0	36	100.0	

**Table 5:** Allele Frequencies Compared to Alcoholic liver disease (ALD)/hepatitis C virus (HCV) Groups—SNP rs4893819 (-1275 G > A).

Allele	ALD *n* (%)		HCV *n* (%)		*p* Value
A/A	**10**	22.7	4	28.6	0.8715
G/G	22	50.0	7	50.0	
G/A	12	27.3	3	21.4	
**Total**	44	100.0	14	100.0	
A/A	10	22.7	4	28.6	0.7245
G/G_G/A	34	77.3	10	71.4	
**Total**	44	100.0	14	100.0	
A/A_G/A	22	50.0	7	50.0	1.000
G/G	22	50.0	7	50.0	
**Total**	44	100.0	14	100.0	
A	32	36.4	11	39.3	0.9568
G	56	63.6	17	60.7	
**Total**	88	100.0	28	100.0	

**Table 6:** Allele frequencies compared clinical data to Alcoholic liver disease (ALD) SNPs.

**Allele**	**ALD - SNP rs35652124 (-214 A ã G)**															
	**M %**	**F %**	**Age**	**MELD**	**ALT**	**AST**	**GGT**	**TB**	**DB**	**IB**	**ALP**	**LDH**	**ALB**	**INR**	**UR**	**CR**
**A/A**	94.45	5.55	53.61 (2.057)	16.54 (1.101)	46.25 (11.02)	76.91 (19.03)	114.75 (21.65)	3.26 (0.3964)	1.20 (0.2407)	2.05 (0.3092)	214.2 (39.73)	460.23 (48.82)	3.12 (0.1612)	1.68 (0.0984)	27.77 (2.699)	1.04 (0.183)
**G/G**	100	0	55.25 (3.304)	16 (0.7559)	51.71 (4.46)	77.34 (13.1)	212 (56.35)	3.43 (0.5941)	1.68 (0.505)	1.79 (0.1932)	191.3 (51.58)	468.6 (57.1)	3.07 (0.1686)	1.48 (0.085)	34.72 (4.387)	0.95 (0.112)
**A/G**	100	0	52.21 (2.924)	17 (1.075)	30.47 (5.188)	39.93 (4.364)	88.34 (27.72)	3.36 (0.5608)	1.42 (0.4408)	2.04 (0.2102)	125.63 (16.44)	421.02 (46.45)	3.25 (0.1566)	1.82 (0.1526)	38.04 (3.144)	0.99 (0.068)
***p* value**	-	-	0.7761	0.8396	0.2224	0.1226	0.0514	0.9742	0.6619	0.7853	0.1928	0.8082	0.7287	0.2295	0.0643	0.9129
**Allele**	**ALD - SNP rs4893819 (-1275 G ã A)**															
	**M %**	**F %**	**Age**	**MELD**	**ALT**	**AST**	**GGT**	**TB**	**DB**	**IB**	**ALP**	**LDH**	**ALB**	**INR**	**UR**	**CR**
**A/A**	88.89	11.11	48.33 (2.809)	16.66 (0.8028)	46.2 (5.275)	60.05 (11.8)	112.63 (39.07)	3.26 (0.439)	1.3 (0.2762)	2 (0.2608)	159.2 (23.81)	421.44 (40.07)	3.01 (0.04)	1.56 (0.0898)	26.76 (5.056)	1.36 (0.321)
**G/G**	100	0	53 (2.282)	16.71 (1.029)	44.87 (8.916)	70.73 (15.51)	118.7 (20.65)	3.24 (0.417)	1.2 (0.2856)	2.03 (0.2483)	151.06 (18.09)	468.6 (47.46)	3.10 (0.149)	1.81 (0.1171)	29.85 (3.431)	0.9 (0.074)
**G/A**	100	0	59 (2.967)	16.86 (1.487)	36 (10.75)	55.67 (15.92)	180 (79.48)	3.71 (0.5114)	1.63 (0.4826)	1.89 (0.3667)	268.3 (79.75)	443.6 (75.63)	3.47 (0.2327)	1.56 (0.1429)	35.45 (5.895)	1.51 (0.107)
***p* value**	-	-	0.0563	0.9946	0.7944	0.8009	0.5107	0.7671	0.7181	0.9516	0.1054	0.8480	0.2425	0.2825	0.6952	0.1259
**Allele**	**ALD - SNP rs6721961 (-178 A ã G)**															
	**M %**	**F %**	**Age**	**MELD**	**ALT**	**AST**	**GGT**	**TB**	**DB**	**IB**	**ALP**	**LDH**	**ALB**	**INR**	**UR**	**CR**
**A/A**	90	10	56.5 (3.212)	16.63 (1.101)	39.5 (7.058)	72.75 (10.37)	158.71 (60.2)	3.64 (0.3599)	1.63 (0.3632)	2.03 (0.3692)	267.62 (68.49)	381.42 (58.18)	3.3 (0.184)	1.5 (0.1257)	37 (4.687)	1.32 (0.269)
**G/G**	100	0	49.71 (1.957)	15 (0.7977)	40.18 (5.564)	58.94 (8.13)	115.7 (21.53)	2.63 (0.3742)	0.93 (0.1771)	1.87 (0.2752)	146.1 (16.06)	471.01 (49.53)	3.07 (0.1274)	1.7 (0.107)	32.04 (3.942)	0.84 (0.051)
**A/G**	100	0	57.14 (2.344)	17.36 (1.002)	43.73 (11.83)	59.85 (21.59)	120.5 (33.98)	3.74 (0.5585)	1.69 (0.4554)	1.92 (0.181)	126.5 (19.45)	470.8 (47.57)	3.18 (0.198)	1.68 (1.1344)	34.19 (2.451)	0.97 (0.086)
***p* value**	-	-	**0.0482**	0.1959	0.9359	0.7991	0.7017	0.1677	0.2381	0.9141	0.0235	0.4332	0.6492	0.5050	0.6612	0.0623

M: male; F: female; MELD: Model for End-Stage Liver Disease; ALT: alanine transaminase; AST: aspartate transaminase; GGT: Gamma-glutamyltransferase; TB: total bilirubin; DB: direct bilirubin; IB: indirect bilirubin; ALP: alkaline phosphatase; LDH: lactate dehydrogenase; ALB: albumin; INR: international normalized ratio; UR: urea; CR: creatinine.

## Supplementary Materials

Supplementary materials can be found at https://www.mdpi.com/1422-0067/20/14/3589/s1.

## Abbreviations

ALD	Alcoholic Liver Disease
ROS	Reactive Oxygen Species
Nrf2	Nuclear erythroid-related factor 2
SNP	Single Nucleotide Polymorphism
NFE2L2	Nuclear erythroid-related factor 2 gene
HCV	Hepatitis C virus
A	Adenine
G	Guanine
PNPLA3	Patatin-like phospholipase domain-containing protein 3
TM6SF2	Transmembrane 6 superfamily member 2
MBOAT7	Membrane-bound O-acyltransferase domain-containing 7
HCC	Hepatocellular carcinoma
NAFLD	Non-alcoholic fatty liver disease
VLDL	Very low-density lipoprotein
ARE	Antioxidant response element
GST	Glutathione-S-transferase
Q10	Coenzyme Q10
QR	NAD(P)Hiquinone oxidoreductase
SOD1	Superoxide dismutase 1
PD	Parkinson’s disease
M	Male
F	Female
MELD	Model for End-Stage Liver Disease
ALT	Alanine transaminase
AST	Aspartate transaminase
GGT	Gamma-glutamyltransferase
TB	Total bilirubin
DB	Direct bilirubin
IB	Indirect bilirubin
ALP	Alkaline phosphatase
LDH	Lactate dehydrogenase
ALB	Albumin
INR	International normalized ratio
UR	Urea
CR	Creatinine
