# Supplementary material for: Polymorphism in the Promoter Region of NFE2L2 Gene Is a Genetic Marker of Susceptibility to Cirrhosis Associated with Alcohol Abuse
Source: Int J Mol Sci. 2019 Jul 23;20(14):3589. doi: 10.3390/ijms20143589 (PMC6678089; doi:10.3390/ijms20143589)
Supplement: Supplementary file 1 [file ijms-20-03589-s001.pdf]

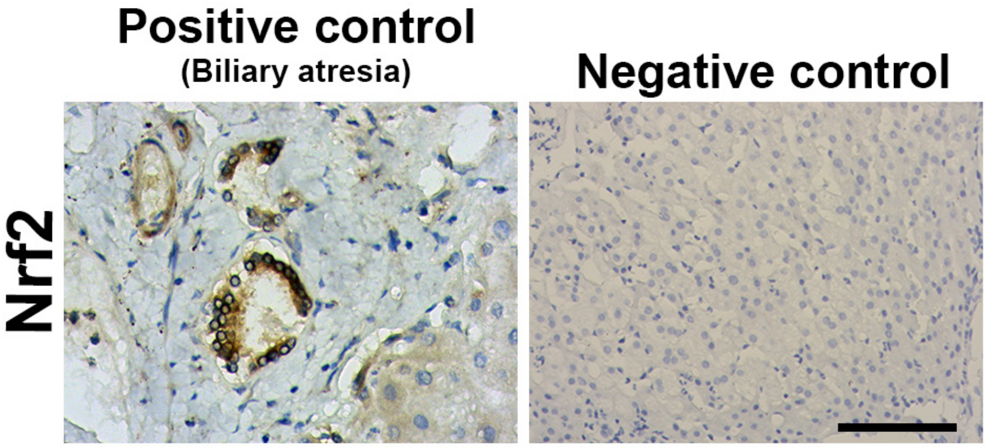

Supplementary figure 1

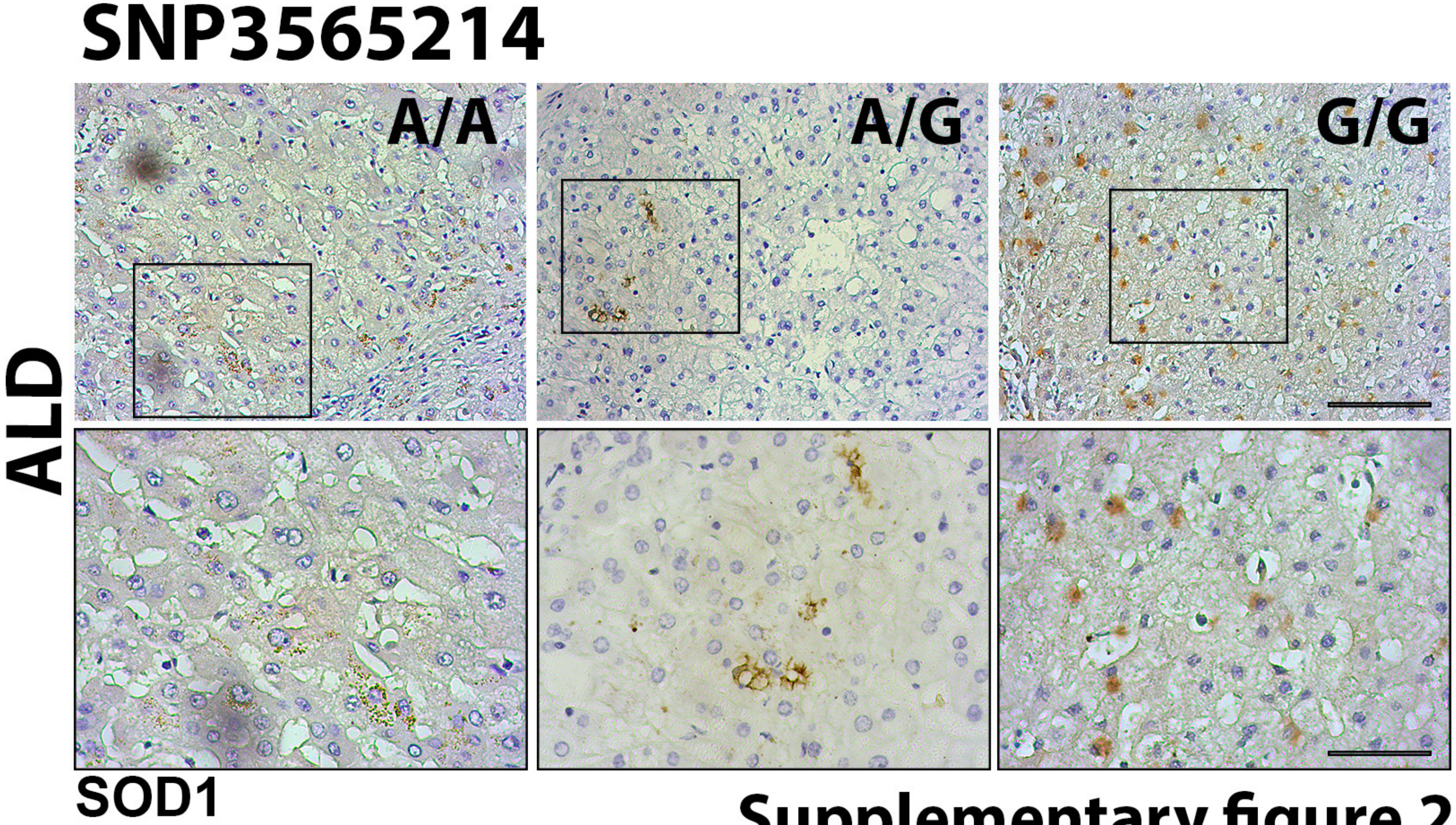

Supplementary figure 2

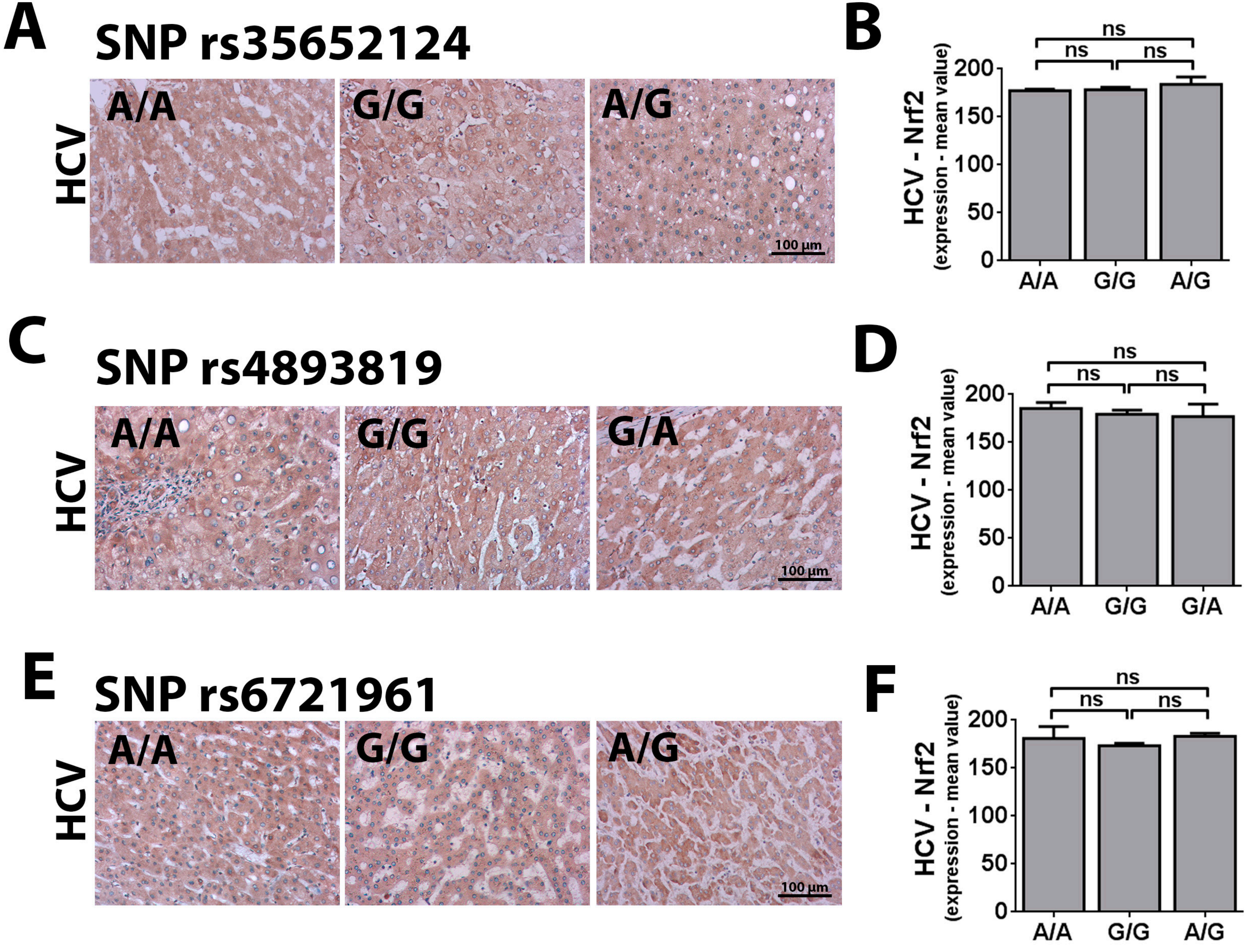

Supplementary figure 3

Supplementary Table 1: Clinical data of the groups

	**ALD (49 patients)**	**HCV (18 patients)**	**Control (21 patients)**
**MALE (%)**	97,68	81,25	25
**FEMALE (%)**	2,32	18,75	75
**AGE (years)**	53,62	57,87	49,5
**MELD**	16,51	14,3	7,75
**ALT (U/mL)**	41,26	98,38	20,5
**AST (U/mL)**	62,82	106,39	39,75
**GGT**	125,96	134,3	64,5
**TB (mg/dL)**	3,37	3,28	0,57
**DB (mg/dL)**	1,42	1,47	0,25
**IB (mg/dL)**	1,98	1,81	0,32
**ALD**	173,59	156,91	110,25
**LDH**	439,25	476,62	-
**ALB**	3,2	3,17	-
**INR**	1,67	1,5	-
**UREA**	33,7	23,9	31,75
**CREATININE**	1,04	0,77	0,91

Supplementary Table 2: allele frequencies compared to clinical data of Alcoholic liver disease (ALD)/hepatitis C virus (HCV) - SNP rs35652124 (-214 A > G)

	**ALD**	**HCV**	***p* value**	**ALD**	**HCV**	***p* value**	**ALD**	**HCV**	***p* value**
	**A/A**	**A/A**		**G/G**	**G/G**		**A/G**	**A/G**	
**MALE %**	94,45	100	-	100	80	-	100	80	-
**FEMALE %**	5,55	0	-	0	20	-	0	20	-
**AGE**	53,61 (2,057)	51 (0)	0,6844	55,25 (3,304)	60,4 (3,01)	0,3101	52,21 (2,924)	57,4 (4,007)	0,3568
**MELD**	16,54 (1,101)	19,5 (4,5)	0,3706	16 (0,7559)	14 (0)	0,2199	17 (1,075)	12 (2,646)	0,0612
**ALT**	46,25 (11,02)	73,5 (3,5)	0,3488	51,71 (4,46)	135,5 (0,5)	**<0,0001**	30,47 (5,188)	75,5 (18,05)	**0,0052**
**AST**	76,92 (19,03)	151 (26,05)	0,1558	77,34 (13,1)	123 (14)	0,1277	39,94 (4,364)	82,75 (21,26)	**0,0093**
**GGT**	114,8 (21,65)	84,5 (7,8)	0,5917	212 (56,35)	85,5 (24,5)	0,2926	88,35 (27,72)	131 (46,69)	0,4427
**TB**	3,272 (0,3964)	4,485 (3,285)	0,4118	3,437 (0,5941)	3,25 (0,85)	0,8826	3,363 (0,5608)	2,88 (1,003)	0,6641
**DB**	1,203 (0,2407)	2,665 (2,565)	0,1717	1,681 (0,505)	1,1 (0,3)	0,5792	1,426 (0,4408)	0,9225 (0,1118)	0,4516
**IB**	2,051 (0,3092)	1,82 (0,72)	0,7818	1,794 (0,1932)	2,15 (0,55)	0,4520	2,049 (0,2102)	1,958 (0,9039)	0,8957
**FALK**	214,2 (39,73)	226,2 (125,8)	0,9153	191,3 (51,58)	102,5 (74,5)	0,4308	125,6 (16,44)	165,7 (52,37)	0,3413
**LDH**	460,2 (48,82)	450,5 (231,5)	0,9478	468,6 (57,1)	394,5 (191,5)	0,6175	421 (46,45)	436,2 (125)	0,8892
**ALB**	3,12 (0,1612)	3,01 (0,19)	0,8012	3,071 (0,1686)	2,9 (0,5)	0,6769	3,258 (0,1566)	3,25 (0,1708)	0,9773
**INR**	1,682 (0,09848)	2,23 (0,26)	0,0636	1,487 (0,08501)	1,33 (0,08)	0,3872	1,824 (0,1526)	1,313 (0,1151)	0,0780
**UREA**	27,77 (2,699)	18,5 (2,5)	0,2168	34,73 (4,387)	26,5 (1,5)	0,3727	38,05 (3,144)	26,25 (1,887)	**0,0494**
**CREATININE**	1,048 (0,1839)	0,92 (0,05)	0,7960	0,9571 (0,1129)	0,85 (0,05)	0,6453	0,9927 (0,06683)	0,78 (0,07439)	0,1020

Supplementary Table 3: allele frequencies compared to clinical data of Alcoholic liver disease (ALD)/hepatitis C virus (HCV) - SNP rs4893819 (-1275 G > A)

	ALD	HCV	*p* value	ALD	HCV	*p* value	ALD	HCV	*p* value
	**A/A**	**A/A**		**G/G**	**G/G**		**G/A**	**G/A**	
**MALE**	88,89	100	-	100	100	-	100	100	-
**FEMALE**	11.11	0	-	0	0	-	0	0	-
**AGE**	48,33 (2,809)	60 (9)	0,1319	53 (2,282)	55 (2,91)	0,6416	59 (2,967)	63 (3)	0,5626
**MELD**	16,67 (0,802)	13,5 (1,5)	0,0996	16,71 (1,029)	12 (1,581)	**0,0406**	16,86 (1,487)	15,5 (1,5)	0,6637
**ALT**	46,2 (5,275)	25 (2)	0,0710	44,87 (8,916)	89,33 (12,33)	**0,0500**	36 (10,75)	110 (26)	**0,0187**
**AST**	60,05 (11,8)	56,5 (13,5)	0,8796	70,73 (15,51)	112 (15,32)	0,2080	55,67 (15,92)	105,5 (31,5)	0,1777
**GGT**	112,6 (39,07)	187,5 (67,5)	0,3748	118,7 (20,65)	105,4 (13,39)	0,7525	180 (79,48)	47,5 (13,5)	0,3968
**TB**	3,263 (0,439)	2,125 (0,375)	0,2148	3,249 (0,417)	2,13 (0,6742)	0,2132	3,71 (0,5114)	4,1 (1,7)	0,7625
**DB**	1,305 (0,276)	0,85 (0,15)	0,4060	1,205 (0,2856)	0,7475 (0,266)	0,4271	1,63 (0,4826)	1 (0,2)	0,4742
**IB**	2,003 (0,260)	1,275 (0,225)	0,1860	2,038 (0,2483)	1,383 (0,4633)	0,2311	1,898 (0,3667)	3,1 (1,5)	0,2846
**FALK**	159,2 (23,81)	227 (94)	0,3164	151,1 (18,09)	129,8 (17,59)	0,5698	268,3 (79,75)	64 (36)	0,2354
**LDH**	421,4 (40,07)	368,5 (199,5)	0,6896	468,6 (47,46)	436,4 (110,4)	0,7648	443,6 (75,63)	419 (216)	0,8909
**ALB**	3,017 (0,040)	3,1 (0,1)	0,3758	3,107 (0,1493)	3,15 (0,263)	0,8940	3,471 (0,2327)	3,1 (0,3)	0,4596
**INR**	1,56 (0,0898)	1,51 (0,09)	0,7779	1,811 (0,1171)	1,378 (0,2028)	0,1017	1,553 (0,1647)	1,385 (0,025)	0,6199
**UREA**	30,93 (3,372)	20,5 (0,5)	0,1413	31,73 (3,067)	24 (2,799)	0,2298	35,71 (4,704)	28,5 (0,5)	0,4606
**CREATININE**	1,313 (0,374)	0,65 (0,01)	0,3693	0,8513 (0,060)	0,8875 (0,037)	0,7695	1,083 (0,0953)	0,75 (0,05)	0,1207

Supplementary Table 4: allele frequencies compared to clinical data of Alcoholic liver disease (ALD)/hepatitis C virus (HCV) - SNP rs6721961(-178 A > G)

	ALD	HCV	*p* value	ALD	HCV	*p* value	ALD	HCV	*p* value
	A/A	A/A		G/G	G/G		A/G	A/G	
**MALE**	90	66,66	-	100	100	-	100	87,5	-
**FEMALE**	10	33,33	-	0	0	-	0	12,5	-
**AGE**	56,5 (3,212)	60,67 (1,202)	0,3493	49,71 (1,957)	40,33 (7,446)	0,1039	57,14 (2,344)	57,5 (2,86)	0,9257
**MELD**	16,63 (1,101)	13 (2,121)	0,1208	15 (0,7977)	11 (2,517)	0,0632	17,36 (1,002)	15,4 (2,731)	0,4122
**ALT**	39,5 (7,058)	110,9 (35,16)	**0,0196**	40,18 (5,564)	15 (1)	0,0993	43,73 (11,83)	88,4 (12,11)	**0,0382**
**AST**	72,75 (10,37)	92 (18,91)	0,3508	58,94 (8,13)	54 (27,62)	0,8146	59,85 (21,59)	117,4 (19,7)	0,1228
**GGT**	158,7 (60,2)	108,8 (21,86)	0,5622	115,7 (21,53)	77 (16,5)	0,4047	120,5 (33,98)	159,6 (94,09)	0,6300
**TB**	3,648 (0,3599)	2,645 (0,6874)	0,1804	2,632 (0,3742)	1,74 (1,18)	0,3497	3,745 (0,5585)	3,64 (1,342)	0,9317
**DB**	1,631 (0,3632)	1,325 (0,6295)	0,6585	0,934 (0,1771)	0,586 (0,4093)	0,3902	1,698 (0,4554)	1,608 (0,9225)	0,9225
**IB**	2,039 (0,3692)	1,32 (0,3656)	0,2358	1,871 (0,2752)	1,153 (0,7764)	0,2868	1,929 (0,181)	2,032 (0,7362)	0,8579
**FALK**	267,6 (68,49)	173,4 (31,02)	0,3748	146,1 (16,06)	130,3 (26,57)	0,6604	126,5 (19,45)	139,7 (55,29)	0,7789
**LDH**	381,4 (58,18)	547,2 (135,2)	0,2128	471 (49,53)	585,5 (0,5)	0,3622	470,8 (47,57)	412,4 (102,8)	0,5645
**ALB**	3,308 (0,184)	3,25 (0,1555)	0,8448	3,075 (0,1274)	2,35 (0,05)	**0,0445**	3,185 (0,198)	3,264 (0,2381)	0,8187
**INR**	1,505 (0,1257)	1,383 (0,1684)	0,5802	1,706 (0,107)	1,245 (0,005)	0,1148	1,685 (0,1344)	1,66 (0,2535)	0,9236
**UREA**	37 (4,687)	22,25 (3,945)	0,0706	32,04 (3,942)	35,33 (8,413)	0,7177	34,19 (2,451)	25 (2,811)	**0,0428**
**CREATININE**	1,326 (0,2696)	0,67 (0,1338)	0,1326	0,8442 (0,051)	1,047 (0,5133)	0,4299	0,9727 (0,086)	0,842 (0,0446)	0,3436
